# Cavitation in a periodontal pocket by an ultrasonic dental scaler: A numerical investigation

**DOI:** 10.1016/j.ultsonch.2022.106178

**Published:** 2022-09-29

**Authors:** You Yu, Mehdi Mahmud, Nina Vyas, Warren R. Smith, Qianxi Wang, A. Damien Walmsley

**Affiliations:** a School of Mathematics, College of Engineering and Physical Sciences, University of Birmingham, Birmingham B15 2TT, UK; b Department of Mathematics, College of Science, Salahaddin University-Erbil, Kurdistan Region, Iraq; c School of Dentistry, College of Medical and Dental Sciences, University of Birmingham, Birmingham B5 7SA, UK

**Keywords:** Dental ultrasonic scalers, Cavitation, Dental cleaning, Finite element method

## Abstract

Periodontal pockets are spaces or holes surrounded by teeth under the gum line. These pockets can become filled with infection-causing bacteria resulting in tissue, bone, and tooth loss. Cavitation produced by the oscillating tip of dental ultrasonic scalers plays a significant role in routine periodontal therapy to clean these areas. Numerical studies were conducted for a scaler vibrating in a periodontal pocket which was simplified to a hole, using ABAQUS based on the finite element method. The simulations consider the three-dimensional, nonlinear, and transient interaction between the vibration and deformation of the scaler tip, the water flow around the scaler and the cavitation formation. The numerical model was validated by comparing results with experimental data for a scaler vibrating in an unbounded liquid, the displacement at the free end of the scaler and the cavitation pattern near the scaler tip displaying excellent agreement. A parametric study for a scaler vibrating in a hole has been carried out in terms of the volume of the hole, the taper ratio (the radius ratio between the circular opening and bottom of the hole), and the immersion depth of the scaler tip in the hole. The amount of cavitation generated is evaluated by the cavitation density (or the void fraction) which is the ratio of the volume of the cavitation occupied in the hole to the total volume of the hole. Numerical results indicate that the cavitation density in the hole increases with the decreasing hole volume and the increasing taper ratio. It is inferred that cleaning effects could be increased if some modifications to the scaler design could be made to increase the blocking effect of the hole during the cleaning process. Cavitation is observed in the hole even if the scaler is placed above the hole and increases with the immersion depth.

## Highlights

• Numerical studies were conducted for a scaler vibrating in a periodontal pocket.
• The scaler deformation, the water flow and the cavitation formation was considered.
• The numerical model was validated by comparison with experiments.
• The cavitation in the hole increases with the proximity of the hole.
• The cavitation in the hole increases with the immersion depth of the scaler tip.

## 1 Introduction

Periodontal pockets are deeper spaces or holes between the gum tissue and the teeth. These pockets are the perfect area for more plaque to accumulate leading to tooth loss. Dental ultrasonic scalers are used in routine periodontal therapy to clean dental plaque biofilms from teeth and dental implants[31]. Cleaning the surface of dental implants has been reviewed and there is no preferred method that is seen as the best treatment[19]. However, none of the studies has considered the use of cavitation alone on the implant surface. Such an approach to cleaning may not lead to significant alteration to the surface being cleaned compared to other procedures[29]and will not require particulate matter as used in air polishing.

**Fig. 1:**
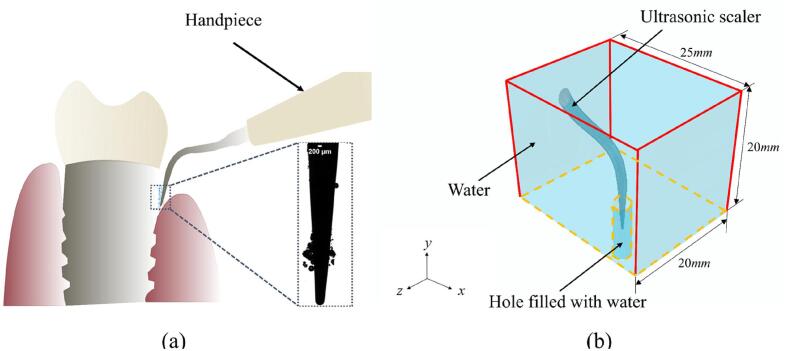
(a) Schematic problem description of ultrasonic scaler tip and its vibration in a periodontal pocket [29], (b) 3D view of the computational domain where the periodontal pocket is simplified to a hole.

Cavitation, the formation and collapse of microbubbles in a liquid, occurs in the cooling water around the tips of ultrasonic scalers [8], [10], [29], [32]. Cleaning effects take place via several mechanisms upon the collapse of cavitation bubbles including microjets formed during microbubble collapse creating localized shear stress on the biofilm [4], [20], [28], cavitation cloud collapse[30]), shock waves emitted during microbubble implosion[22], micro-streamers, acoustic streaming in the bulk fluid and microstreaming around individual cavitation bubbles as they oscillate[17]. This cavitation may be used to clean dental biofilm via a non-touching technique and would be helpful in difficult-to-reach areas of the mouth such as within periodontal pockets, root furcation or on artificial surfaces such as dental implants. It has been confirmed by previous studies that the cavitation occurs around dental scaler tips and aids the cleaning process [8], [24], [25]. Increasing the cavitation generated by an oscillating scaler tip will lead to novel clinical approaches to the cleaning process. However, there is currently little understanding of the cavitation behaviour when the scaler tip is in a confined space, e.g., a scaler in a periodontal pocket as shown in Fig. 1a[29].

We will conduct numerical studies for an ultrasonic dental scaler tip vibrating in the water in a periodontal pocket which is simplified to a hole and consider the cavitation generation around the scaler tip. The amount of cavitation generated is evaluated by the cavitation density (or the void fraction), the ratio of the volume of the cavitation occupied in the hole to the total volume of the hole. The numerical simulations can provide an insight into how cavitation may be affected by the hole volume, taper ratio, and immersion depth of the scaler tip. The information will indicate the cleaning effects during the dental cleaning process. The numerical capability can also be used to optimize the scaler design and operation parameters. This study is for the fluid being pure water. Dissolved gases/substances in the solvent will enhance the growth of bubbles due to rectified diffusion. Vyas et al.[27] observed significantly improved biofilm removal using cavitation from a dental ultrasonic scaler vibrating in carbonated water.

In the numerical study, the deformation of the scaler tip is modelled by isotropic linear elastic theory, the water flow induced is modelled by the linear potential flow theory in terms of pressure, and their interaction is modelled through the kinetic and dynamic boundary conditions at their interface. Hydrodynamic cavitation is a phenomenon in which the static pressure of a liquid reduces to below the liquid's vapour pressure, leading to the formation of small vapor-filled bubbles in the liquid[36], [14]. As the detailed micro-processes of cavitation generation remain elusive[15], the one-fluid, cut-off cavitation model is employed to model the generation of vaporous cavitation in this paper[33], [21], [3], [34], [18]. It solves the single phased governing equations used to model two separated phases of the fluid (e.g., water and vapour), and treats the cavitation region as a homogenous single-phase region of constant total pressure equal to the vapour pressure of water once the cavitation criterion is met[12]. Wardlaw & Luton[33]noticed that the pressure cut-off has little impact on the numerical solutions if it is positive and small compared to 1 bar. Although the vapour phase of the cavitation is not explicitly considered and is evaluated by the volume of region where the pressure falls below the saturated vapour pressure, the model has approximated global cavitation zones that were predicted by the cavitation models which include phase transition[16], [35].

The numerical model is described in section 2, and it is validated in section 3 by comparing numerical results with experimental data for the displacement at the free end of the scaler and the cavitation pattern near the scaler tip for a scaler operating in unbounded water. In section 4, the formation of cavitation due to a scaler vibrating in a hole is analysed, and parametric analyses have been carried out to study the effects of the hole volume, the taper ratio, and the immersion depth of the scaler tip on the cavitation generated around the scaler tip in the hole. Finally, the summary and conclusions are outlined in section 5.

## 2 Numerical model

### 2.1 Problem description

**Fig. 2:**
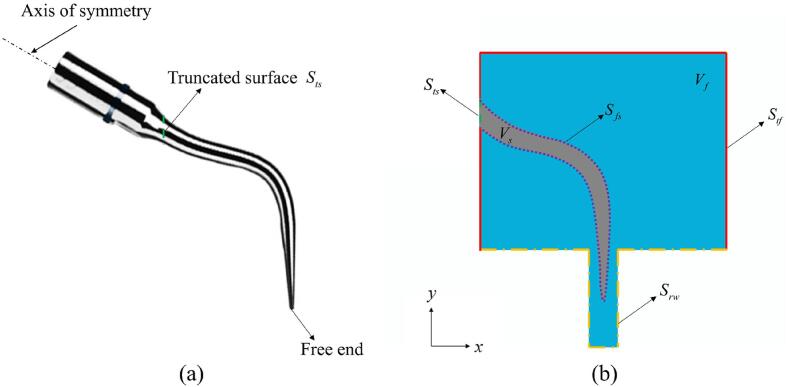
The illustration of (a) the scaler with the truncated surface $S_{ts}$, and (b) the cross section of the computational domain at the *Oxy*-plane indicating the position of the displacement boundary condition at $S_{ts}$, the fluid–structure interface $S_{fs}$, the rigid wall surface $S_{rw}$ and the truncated water planes $S_{tf}$.

Considering a dental scaler tip vibrating inside a periodontal pocket (hole) filled with water, as illustrated in Fig. 1b. A numerical model is described as follows for the three-dimensional, non-linear, and transient interaction between the scaler vibration, the liquid flow, and the cavitation development. It is assumed that the configuration is symmetric in a vertical plane, which is chosen to be the *Oxy*-plane with the *y*-axis vertical. The cubic part of the computational domain is 25 mm×20 mm×20 mm in *x*,*y*, and *z* directions, respectively. The section near the circular cylinder base of the scaler is much thicker than the free end of the scaler and does not deform significantly [18]. We thus truncate the part that does not deform with the truncated scaler face sts as shown in Fig. 2a, where a prescribed one-dimensional harmonic oscillation along the axis of symmetry of the circular cylinder base is defined. Its displacement is *A*sin(*ωt*) where *A* is the amplitude and *ω* is the angular frequency. The choice of the truncated computational domain size is validated in section 3.2. The vibration of the scaler tip generates pressure waves in the water which results in cavitation formation.

As shown in Fig. 2b, the truncated part of the scaler is assumed to be an elastic structure with the domain $V_{s}$ and its surface *S*_*s*_ = *S*_*fs*_ ∪ *S_ts_* where *S*_*fs*_ is the fluid–structure interface within the truncated computational domain, and $S_{ts}$ is the truncated scaler surface. The truncated water domain used for numerical simulations is denoted as $V_{f}$ bounded by the interface surface $S_{fs}$, the rigid wall surface $S_{rw}$ consisting of the bottom of the container and the hole wall, and the five truncated water planes $S_{tf}$ of the cubic part of the computational domain. The boundary conditions imposed on these surfaces are introduced in the following sections.

### 2.2 Structure modelling

Suppose the structure (scaler) under the action of the body force $f_{b}$ obeys the isotropic linear elastic theory. Its displacement $u_{s}$ satisfies the linear momentum equation[2].

(1) $$\nabla \cdot \sigma_{s}+\rho_{s}f_{b}=\rho_{s}{\overset{¨}{u}}_{s},$$

where ∇ represents the nabla operator, **σ***_s_* and *ρ*_s_ are the stress tensor and density of the structure, respectively, and **ü***_s_* is the second order time derivative of **u**_*s*_ denoting the acceleration.

Introducing an arbitrary variation displacement field $\delta u_{s}$, and integrating equation (1) over the structural domain $V_{s}$, an equivalent weak form for the linear momentum equation of the structure can be obtained.

(2) $$\underset{V_{s}}{\int }\delta u_{s}\cdot \left[\nabla \cdot \sigma_{s}+\rho_{s}\left(f_{b}-{\overset{¨}{u}}_{s}\right)\right]dV=0.$$

Using the Gauss theorem with the gradient identity for the first term on the left side, and neglecting all body forces $f_{b}$ (such as damping, gravity) and keeping the fluid pressure and surface traction gives us.

(3) $$\underset{V_{s}}{\int }\delta \varepsilon \overset{˙}{\cdot }\sigma_{s}dV+\underset{V_{s}}{\int }\rho_{s}\delta u_{s}\cdot {\overset{¨}{u}}_{s}dV+\underset{S_{fs}}{\int }p_{s}\delta u_{s}\cdot ndS-\underset{S_{ts}}{\int }\delta u_{s}\cdot tdS=0,$$

where δε is the strain variation that is compatible with $\delta u_{s}$, n is the unit outward normal to the structure surface (the inward normal to the fluid at the boundary), $p_{s}$ is the pressure acting on the fluid-structural interface $S_{fs}$, and t is the surface traction, which is determined by the prescribed displacement at $S_{ts}$.

### 2.3 Acoustic modelling and cavitation

Assume that the water flow induced by the vibrating scaler tip is inviscid, compressible and irrotational. The momentum equation of the flow experiencing velocity-dependent momentum losses can be expressed as[13], [2], [1], [37].

(4) $$\nabla p_{f}+\gamma {\overset{˙}{u}}_{f}+\rho_{f}{\overset{¨}{u}}_{f}=0,$$

with the linear constitutive behaviour of the liquid,

(5) $$p_{f}=-K_{f}\nabla \cdot u_{f},$$

where γ is the volumetric drag which is neglected in our calculations as it is small compared with the fluid inertia, $p_{f}$ is the pressure in the fluid, $u_{f},{\overset{˙}{u}}_{f}$ and ${\overset{¨}{u}}_{f}$ are the displacement, velocity and acceleration of the fluid particle, respectively, $\rho_{f}$ is the density of the fluid, and $K_{f}$ represents the bulk modulus of the fluid.

To obtain the partial differential equation used in direct integration transient analysis in terms of pressure$p_{f}$, equation (4) is divided by $\rho_{f}$ and the divergence is evaluated with respect to x, where x is the spatial position. In the absence of the volumetric drag, the result is then combined with the time derivatives of equation (5) which results in.

(6) $$\frac{1}{K_{f}}{\ddot{p}}_{f}-\nabla \cdot \left(\frac{1}{\rho_{f}}\nabla p_{f}\right)=0,$$

where ${\ddot{p}}_{f}$ is the second order derivative of $p_{f}$ in time t. This is the linear wave equation for the pressure field $p_{f}$.

Introducing an arbitrary variation field δp, and integrating equation (6) over the whole fluid field $V_{f}$, an equivalent weak form for the equation of motion of the fluid can be obtained as follows:

(7) $$\int_{V_{f}}\left(\frac{1}{K_{f}}{\ddot{p}}_{f}-\nabla \cdot \frac{\nabla p_{f}}{\rho_{f}}\right)\delta pdV=0.$$

Green’s theorem allows this to be rewritten as.

(8) $$\underset{V_{f}}{\int }\left(\frac{1}{K_{f}}{\overset{¨}{p}}_{f}\delta p+\frac{1}{\rho_{f}}\nabla \delta p\cdot \nabla p_{f}\right)dV+\underset{S_{rw}+S_{fs}+S_{tf}}{\int }T\left(x\right)\delta pdS=0,$$

(9) $$T\left(x\right)=-\frac{n\cdot \nabla p_{f}}{\rho_{f}}=n\cdot {\overset{¨}{u}}_{f},$$

where the boundary traction $T\left(x\right)$ is equal to the inward acceleration of the particles of the fluid when neglecting the volumetric drag.

Two boundary conditions for the fluid part ($S_{rw}$ and $S_{tf}$) except for the interface of fluid–structure $S_{fs}$ should be prescribed. $S_{rw}$ represents the rigid immobile wall where the acceleration of the particles ${\overset{¨}{u}}_{f}=0$, and we apply this boundary condition by prescribing.

(10) $$T_{rw}\left(x\right)=n\cdot {\overset{¨}{u}}_{f}=0.$$

The non-reflective boundary at the truncated water planes $S_{tf}$.

(11) $$T_{tf}\left(x\right)=-\frac{n\cdot \nabla p_{f}}{\rho_{f}}=-\frac{\cos(\theta )}{\sqrt{\rho_{f}K_{f}}}{\overset{˙}{p}}_{f},$$

where θ is the angle between the sound wave and the normal to the truncated boundary surface.

We assume that cavitation happens instantaneously when the absolute pressure in the liquid is smaller than a threshold $p_{c}$. After considering cavitation, the pressure $p_{f}$ in the liquid follows from.

(12) $$p_{f}=\max\left\{p_{f},p_{c}\right\}.$$

### 2.4 Fluid-structure interaction

Fluid-structure interaction is modelled through the kinetic and dynamic boundary conditions at their interface $S_{fs}$. We apply the kinetic boundary condition, by equating displacement of the fluid and structure.

(13) $$n\cdot u_{s}=n\cdot u_{f},T_{fs}\left(x\right)=n\cdot {\overset{¨}{u}}_{s}.$$

The dynamic boundary condition is adopted by keeping the stress continuous at the interface.

(14) $$n\cdot \sigma_{s}=n\cdot \sigma_{f},$$

where $\sigma_{f}$ and $\sigma_{s}$ are the Cauchy stress tensors in the fluid and structure respectively.

## 3 Numerical validation

The water parameters are chosen as follows: the density $\rho_{f}=1000\;kg/m^{3}$, the bulk modulus $K_{f}\;=\;\rho_{f}c^{2}\;=\;2140\;\text{MPa}$, where c is the speed of sound in water and the cavitation pressure threshold $p_{c}\;=\;2300\;\text{Pa}\;$, which is the saturated vapour pressure for water at room temperature of 20 °C. The scaler (Tip 10P, Acteon Group, USA) is made of stainless steel with the density $\rho_{s}=\;8000\;{\text{kg/m}}^{3}\;$, the Young's modulus E=224 GPa, the Poisson’s ratio ν=0.3 [5], and the yield strength $\sigma_{\mathrm{ys}}=\;215\;\text{MPa}$. The amplitude and the angular frequency of the harmonic oscillation of the truncated end of the scaler are chosen as A=0.01mm and ω=31 kHz which could be used for a clinical setting, respectively.

The computational domain is meshed with unstructured tetrahedral meshes with varying densities to fit the computational domain as well as to save CPU time. For the structural part with 0.1 million elements, the mesh size ranges from 0.01mm near the scaler tip where a higher displacement occurs to 0.06mm near the truncated surface of the scaler. For the fluid part, the mesh size is 0.01mm in the hole that is the region of our interest in this study, 0.02mm on the fluid-structural interface and 1.00mm on the bottom of the cuboid. With the sizes mentioned above, the total element number for the fluid domain is approximately 12 million.

### 3.1 Mesh convergence tests

**Fig. 3:**
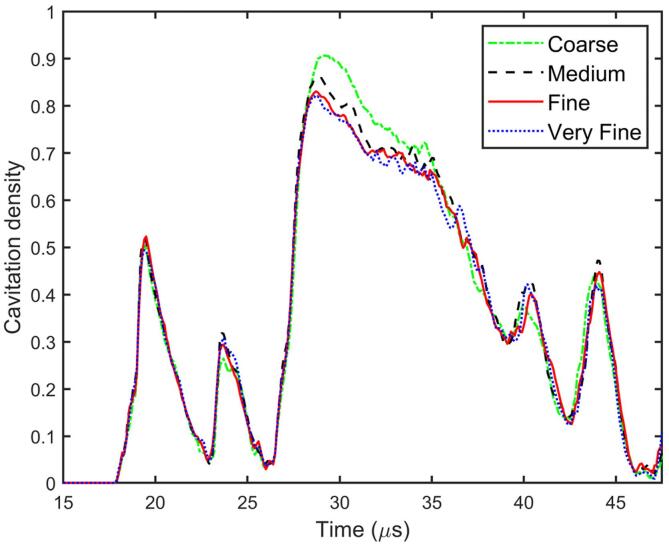
Mesh convergence tests in terms of the time history of the cavitation density within the hole. Element numbers are 2 million, 8 million, 12 million, 16 million for coarse, medium, fine, and very fine mesh, respectively. The hole is a circular cylinder shape with radius r=1.5mm, depth H=10.0mm and the immersion depth of the scaler tip in the hole h=5.0mm. The amplitude A and the angular frequency ω of the harmonic oscillation of the scaler handpiece are 0.01mm and 31kHz. Other parameters used in the calculations are $\rho_{f}\;=\;1000\;{\text{kg/m}}^{3}$, $K_{f}\;=\;\rho_{f}c^{2}\;=\;2140\;\text{MPa}$, $p_{c}\;=\;2300\text{ Pa}$, $\rho_{s}=\;8000\;{\text{kg/m}}^{3}$, E=224GPa, ν=0.3, and $\sigma_{\mathrm{ys}}=\;215\;\text{MPa}$.

The convergence tests are carried out for various element numbers (coarse: 2 million; medium: 8 million; fine: 12 million; very fine: 16 million), with the results displayed in Fig. 3 for the time history of the cavitation density in the hole. The computational domain with the radius of the circular cylinder hole being 1.5 mm and the depth being 10 mm is adopted. The results for the two higher resolutions are close. Therefore, the mesh resolution for the fine mesh with 12 million elements as described above is adopted for all subsequent calculations.

### 3.2 The truncated computational domain

**Fig. 4:**
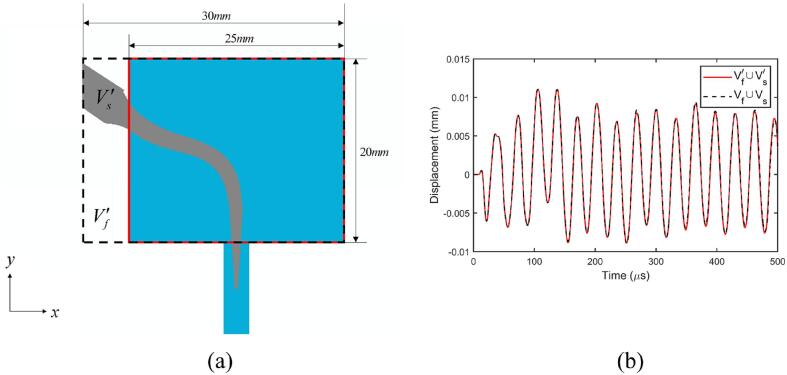
(a) Illustration of the two truncated computational domains at *Oxy*-plane with different sizes. (b) Comparison of the displacement of the free end of the scaler tip along the *x*-axis for the computational domains $V_{f}\cup V_{s}$ and $V_{f}^{\prime }\cup V_{s}^{\prime }.$ Other parameters used in the calculations are the same as in Fig. 3.

To test if the truncated computational domain is sufficient, we will compare the computational results with two truncated domains, denoted as $V_{f}\cup V_{s}$ as showed in Fig. 1b, and a larger computational domain denoted as $V_{f}^{\prime }\cup V_{s}^{\prime }$ as shown in Fig. 4a.

The results compared are the displacement of the scaler tip and the time-averaged cavitation density in the hole over the simulation time 500μs. With a 20 % difference in volume, the displacements for the two fluid domains are very close (Fig. 4b) and the time-averaged cavitation is 0.319 for $V_{f}\cup V_{s}$ and 0.317 for $V_{f}^{\prime }\cup V_{s}^{\prime }$. Therefore, the smaller truncated computational domain $V_{f}\cup V_{s}$ is suitable and chosen for all remaining calculations to save CPU time.

### 3.3 Comparisons with experimental results

We validate the computational results with experiments for the scaler tip vibrating in an unbounded expanse of water, as there are available experimental results for this case. Experimental high-speed images were acquired of an ultrasonic scaler tip vibrating in a water tank using a previously described setup[24]. Briefly, a P5 Newtron XS dental ultrasonic scaler (Satelec, Acteon, France) was used in conjunction with Tip 10P to generate the cavitation around the scaler. Imaging was done in brightfield mode using a high-speed camera (Fastcam mini AX200, Photron, Japan). The scaler position was fixed by attaching it to an *xyz* translation stage (PT3, Thorlabs Inc, NJ, USA) and a high-precision rotation mount (PRO1/M, Thorlabs Inc, NJ, USA). The axial rotation of the scaler tip was maintained during experiments.

**Fig. 5:**
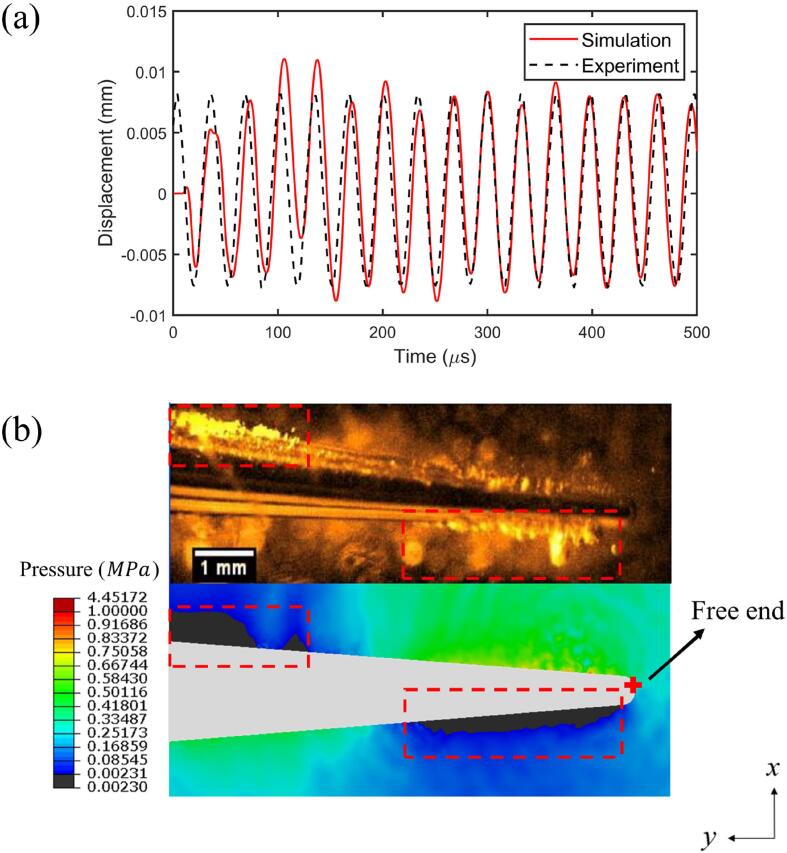
Comparisons with experiments in terms of (a) the displacement of the free end of the scaler tip denoted by “+” in Fig. 4b along the *x*-axis and (b) the cavitation pattern (black area) around the scaler tip at t=348.5μs in the simulation on *Oxy*-plane and in the experiment. Parameters used in the calculations are the same as in Fig. 3.

We compare the computational results with experiments in terms of the displacement of free end of the scaler tip denoted by “+” in Fig. 5b as well as the cavitation pattern around the scaler tip. The boundary condition for the surface *S_rw_* of the fluid domain in Fig. 2b is set as the non-reflective boundary (equation (11)) to satisfy the equivalent infinite fluid domain in the experiment. Fig. 5a depicts the comparison of the scaler tip displacement in the global *x*-direction. For the first few cycles of oscillation, the computational results are associated with some transient oscillations as the initial conditions were set as the quiescent water flow, whereas the experimental data were recorded after many cycles with repeated steady oscillation. After the first few cycles, the displacement of the computational results rapidly stabilizes and agrees well with the experimental data.

Fig. 5b displays the contours of the pressure in the fluid where the predicted area of cavitation is in black at t=348.5μs and the corresponding experimental image of the cavitation zone near the straight part of the vibrating scaler tip [24]). The computational results display-two cavitation zones, shown in the dashed rectangles, one is beneath the tip and near the tip end and the other is above the tip and away from the tip end. The present model predicts the main cavitation zones well. Some other bubbles can also be observed in the experiment from Fig. 5a. This discrepancy could be due to the inaccuracy of the present model and/or the pre-existed air bubbles that grow due to rectified diffusion[9], [23].

## 4 Results and discussion

Parametric study was carried out to investigate the cavitation density in the hole in terms of the hole volume, the taper ratio of the hole, and the immersion depth of the scaler tip. The depth of the holes in our simulations is 10 mm considering advanced periodontitis where the periodontal pocket depth can reach 10 mm or deeper[7]. In consideration of safety in a clinical setting, the vibrating scaler should not touch the tooth or the gum tissue. Therefore, a wide range of the hole size and the immersion depth corresponding to the safety concerns has been adopted in our simulations which will be provided in the following sections. The simulation time is 500μs for each case and the time-averaged cavitation density is evaluated for comparisons.

### 4.1 Cavitation around a scaler tip

**Fig. 6:**
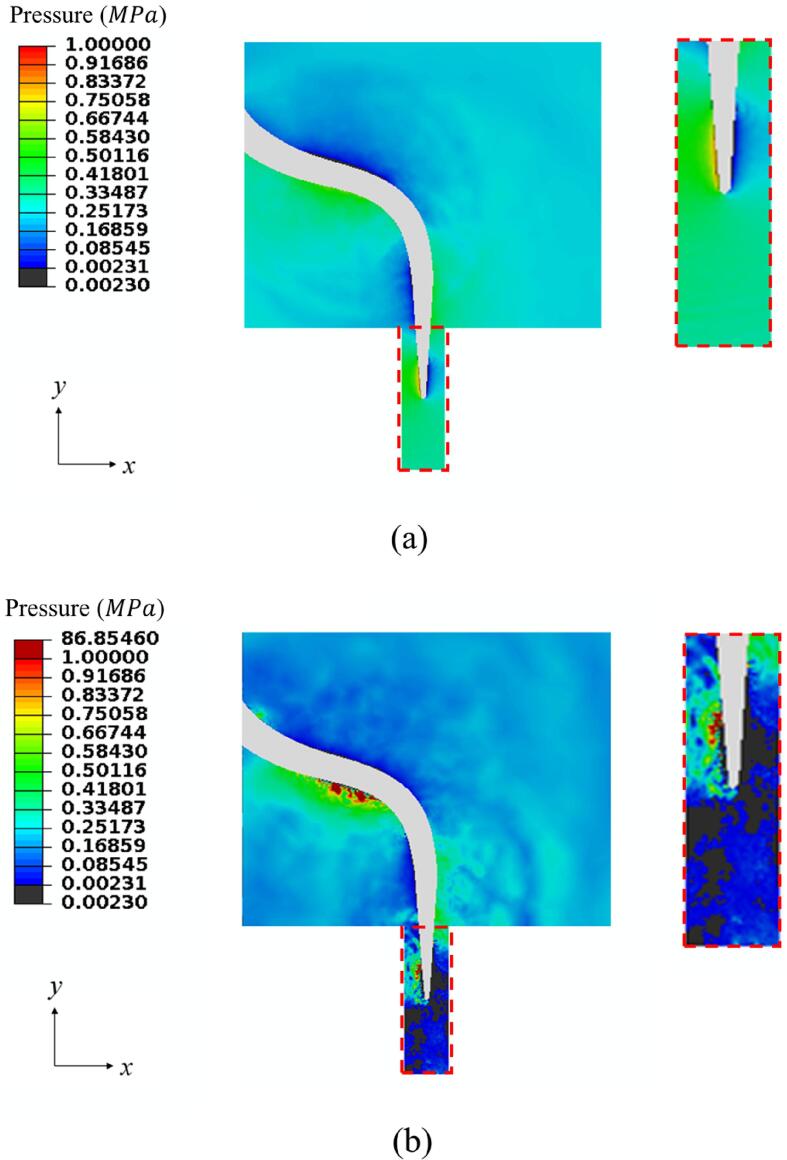
Pressure contours showing the cavitation pattern for a scaler vibrating in (a) an unbounded infinite domain and (b) a hole at t=399.0μs. The black area denotes the area of cavitation. Other parameters used in the calculations are the same as in Fig. 3.

Fig. 6 compares the pressure contours for a scaler tip vibrating (a) in an unbounded infinite domain and (b) in a hole. For a scaler tip vibrating in the infinite domain, the pressure contour is only shown within the corresponding liquid domain for the scaler tip oscillating in a hole. The results demonstrate clearly that much more cavitation is generated around the scaler tip vibrating in a hole. The average energy of acoustic wave per unit volume e is related to the pressure wave amplitude $p_{f}$ by [11].

(15) $$e=\frac{p_{f}^{2}}{2\rho_{f}c^{2}}.$$

For the scaler tip placed in a hole with rigid boundaries, more ultrasonic energy will be confined in the hole in the form of pressure waves with higher amplitude, and thus the pressure in the liquid is more likely to be lower than the vapour pressure resulting in the higher likelihood of the cavitation formation in the hole.

### 4.2 Effects of the hole volume

**Fig. 7:**
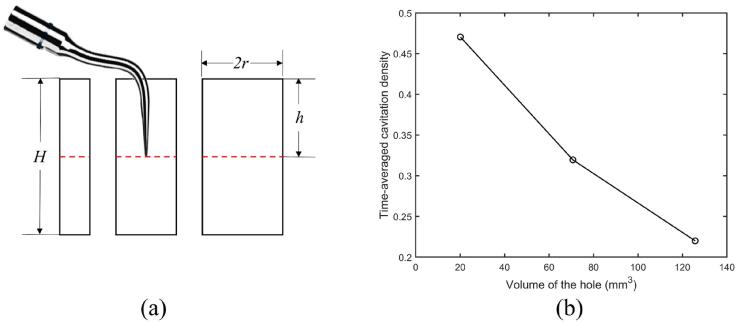
(a) Schematic diagram for a dental scaler vibrating in holes with the volume being approximately 20.1, 70.7, 125.7 ${\text{mm}}^{3}$ (from left to right). (b) Time-averaged cavitation density in the hole versus the volume of the hole. The dimensionless immersion depth of the scaler tip in the hole is 0.50, where the depth is *H* and other parameters used in the calculations are the same as in Fig. 3.

Fig. 7a shows the schematic diagram for a dental scaler vibrating at dimensionless immersion depth h/H of 0.50 in circular cylinder-shaped holes with the depth of the hole *H* being 10.0 mm and the radius *r* being 0.8, 1.5 and 2.0 mm, respectively. The volume of each hole is approximately 20.1, 70.7, 125.7 $mm^{3}$ respectively.

Fig. 7b shows that the time-averaged cavitation density increases as the hole volume decreases. As the time-averaged energy introduced into the fluid in the hole from the vibrating scaler is constant, the ultrasonic energy per unit volume increases with the hole volume decreasing. This results in stronger ultrasonic pressure waves in the hole and produces more cavitation in the hole.

### 4.3 Effects of the taper ratio

**Fig. 8:**
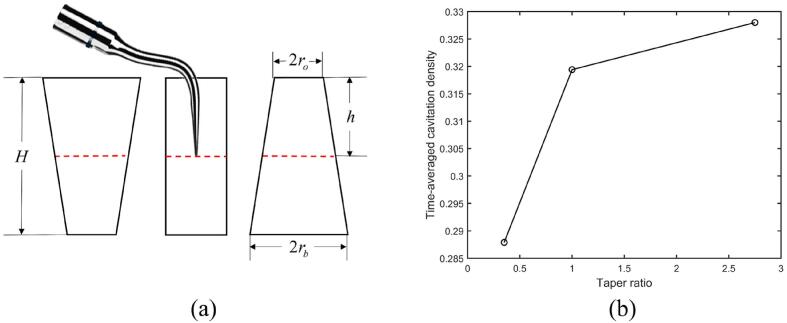
(a) Schematic diagram for a dental scaler vibrating in holes with the depth *H* being 10.0 mm and the volume being 70.7 ${\text{mm}}^{3}$. The taper ratio *η* is 0.35, 1.00 and 2.75 (from left to right). (b) Time-averaged cavitation density in the hole versus the taper ratio of the hole. The dimensionless immersion depth of the scaler tip in the hole is 0.50, and other parameters used in the calculations are the same as in Fig. 3.

The taper ratio is defined as $\eta \;=\;r_{o}/r_{b}$, where ${\text{r}}_{\text{o}}$ and ${\text{r}}_{\text{b}}$ are the radius of the circular opening and bottom of the hole, respectively. Fig. 8a shows the schematic diagram for a dental scaler vibrating at dimensionless immersion depth *h*/*H* of 0.50 in holes with the depth *H* being 10.0 mm and the volume being 70.7 ${\text{mm}}^{3}$. The taper ratio η is 0.35, 1.00 and 2.75, respectively.

Fig. 8b shows that the time-averaged cavitation density increases with the taper ratio. The smaller opening of the hole for the larger taper ratio reduces the wave propagation and associated energy transport outside the hole. The larger ultrasonic energy retained in the hole results in stronger pressure waves, and thus more cavitation in the hole is generated. If a scaler tip is equipped with a lid to seal the hole opening, more cavitation will be generated in the hole, and this will enhance cleaning effects.

### 4.4 Effects of the immersion depth

**Fig. 9:**
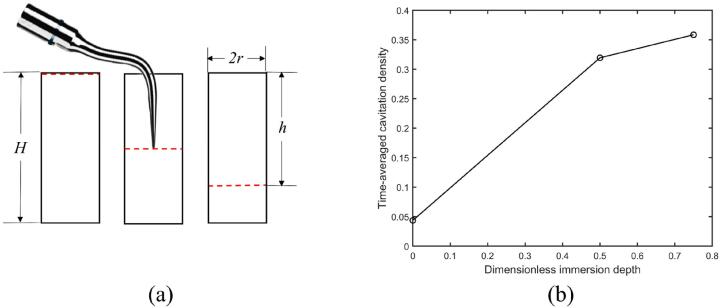
(a) Schematic diagram for a dental scaler vibrating in holes with the volume being 70.7 ${\text{mm}}^{3}$ and the taper ratio being 1.00. The dimensionless immersion depths are 0.00, 0.50, 0.75 (from left to right). (b) Time-averaged cavitation density in the hole versus the dimensionless immersion depth of the scaler tip. Other parameters used in the calculations are the same as in Fig. 3.

Fig. 9a shows the schematic diagram for a dental scaler vibrating in a hole with the depth of the hole *H* being 10.0 mm, the volume being 70.7 ${\text{mm}}^{3}$ and the taper ratio being 1.00 at the dimensionless immersion depth *h*/*H* of 0.00, 0.50 and 0.75, respectively*.*

Fig. 9b shows that the time-averaged cavitation density increases as the immersion depth of the scaler tip increases. With a larger immersion depth, more energy is introduced into the liquid in the hole leading to stronger pressure waves in the hole. Consequently, cavitation is more likely to be generated.

**Fig. 10:**
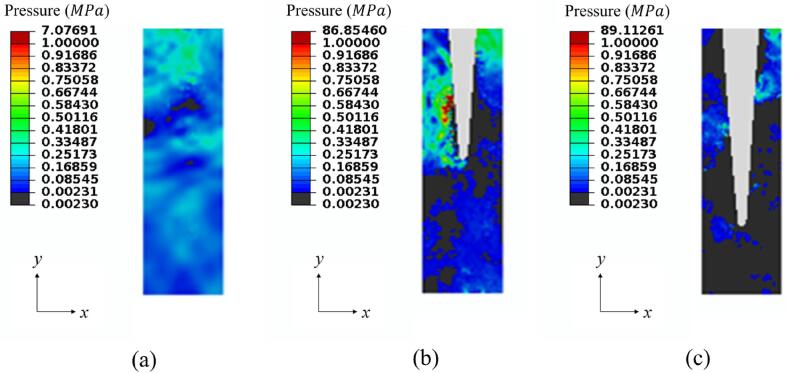
Pressure contours at t=399.0μs for cases with dimensionless immersion depths being (a) 0.00, (b) 0.50, and (c) 0.75 on the *Oxy-*plane of the hole. The black area denotes the area of cavitation. Other parameters used in the calculations are the same as in Fig. 3.

Fig. 10 shows pressure contours in the three cases with different immersion depths at t=399.0μs when the oscillation is relatively stable. Cavitation can be observed in the hole when the immersion depth is 0.00. This means cleaning effects exists even if the scaler is placed just above the hole. The cavitation density and the cleaning effects increases significantly with the immersion depth.

## 5 Summary and conclusions

A numerical study has been undertaken for the cavitation generated by a dental ultrasonic scaler tip oscillating in a periodontal pocket using a coupled acoustic-structural finite element method. As the one-fluid, cut-off cavitation model is employed, cavitation is evaluated by the volume of the region where the pressure falls below the cavitation threshold. The comparison shows that the computational results agree well with experiments in terms of the size and location of the cavitation zones for a scaler tip oscillating in an unbounded expanse of water. The periodontal pocket is simplified to a hole and a parametric study has been performed to investigate the effects of the volume of the hole, the taper ratio η and the immersion depth of the scaler tip on the time-averaged cavitation density in the hole. Based on the numerical results, it can be concluded that:

More cavitation around a scaler tip is generated when it is vibrating in a hole, and the cavitation density increases as the hole volume decreases. This is because the ultrasound energy per unit volume increases inversely with the hole volume.
The cavitation density increases with the taper ratio as more energy remains in the hole due to the increased blocking effect. The effects of cleaning should be improved with the addition of a lid added to the scaler tip to cover the hole opening.
Cavitation forms in the hole even if the scaler is placed above the hole. The cavitation density increases significantly with the immersion depth of the scale tip in the hole.
The cavitation density varies when the scaler tip is put in periodontal pockets with various shapes and immersion depths. The increase of cavitation density may result in increased cleaning efficiency. In this regard, the numerical model developed in this study can aid in further optimization studies that allow the ultrasonic scaler to be used in a non-touching technique in a more efficient way.

## CRediT authorship contribution statement

**You Yu:** Conceptualization, Methodology, Formal analysis, Data curation, Investigation, Visualization, Writing – original draft. **Mehdi Mahmud:** Validation. **Nina Vyas:** Resources. **Warren R. Smith:** Supervision, Writing – review & editing. **Qianxi Wang:** Supervision, Writing – review & editing, Funding acquisition. **A. Damien Walmsley:** Supervision, Writing – review & editing, Funding acquisition.

## Declaration of Competing Interest

The authors declare that they have no known competing financial interests or personal relationships that could have appeared to influence the work reported in this paper.

## Data Availability

Data will be made available on request.
